# Selective Serotonin Reuptake Inhibitor Use in Pregnancy and Protective Mechanisms in Preeclampsia

**DOI:** 10.1007/s43032-022-01065-z

**Published:** 2022-08-19

**Authors:** Julie A. Vignato, S. Banu Gumusoglu, Heather A. Davis, Sabrina M. Scroggins, Wendy S. Hamilton, Debra S. Brandt, Gary L. Pierce, Boyd A. Knosp, Donna A. Santillan, Mark K. Santillan

**Affiliations:** 1 College of Nursing, University of Iowa, Iowa City, IA 52242, USA; 2 Department of Obstetrics and Gynecology, Carver College of Medicine, University of Iowa, Iowa City, IA 52242, USA; 3 Institute for Clinical and Translational Science, Carver College of Medicine, University of Iowa, Iowa City, IA 52242, USA; 4 College of Liberal Arts and Sciences, Department of Health and Human Physiology, University of Iowa, Iowa City, IA 52242, USA; 5 Francois M. Abboud Cardiovascular Research Center, Iowa City, IA 52246, USA; 6 Interdiciplinary Program in Molecular Medicine, University of Iowa, Iowa City, IA 52242, USA

**Keywords:** Depression, Symptoms, Copeptin, Pregnancy, Preeclampsia, Selective serotonin reuptake inhibitors

## Abstract

Depression and preeclampsia share risk factors and are bi-directionally associated with increased risk for each other. Despite epidemiological evidence linking selective serotonin reuptake inhibitors (SSRIs) in pregnancy to preeclampsia, serotonin (5-HT) and vasopressin (AVP) secretion mechanisms suggest that SSRIs may attenuate preeclampsia risk. However, there is a need to clarify the relationship between SSRIs and preeclampsia in humans to determine therapeutic potential. This retrospective cohort study included clinical data from 9558 SSRI-untreated and 9046 SSRI-treated pregnancies. In a subcohort of 233 pregnancies, early pregnancy (< 20 weeks) maternal plasma copeptin, an inert and stable AVP prosegment secreted 1:1 with AVP, was measured by enzyme-linked immunosorbent assay. Diagnoses and depression symptoms (Patient Health Questionnaire-9 [PHQ-9]) were identified via medical records review. Descriptive, univariate, and multivariate regression analyses were conducted (α = 0.05). SSRI use was associated with decreased preeclampsia after controlling for clinical confounders (depression severity, chronic hypertension, diabetes, body mass index, age) (OR = 0.9 [0.7–1.0], *p* = 0.05). Moderate-to-severe depression symptoms were associated with significantly higher copeptin secretion than mild-to-no depression symptoms (240 ± 29 vs. 142 ± 10 ng/mL, *p* < 0.001). SSRIs significantly attenuated first trimester plasma copeptin (78 ± 22 users vs. 240 ± 29 ng/ml non-users, *p* < 0.001). In preeclampsia, SSRI treatment was associated with significantly lower copeptin levels (657 ± 164 vs. 175 ± 134 ng/mL, *p* = 0.04). Interaction between SSRI treatment and preeclampsia was also significant (*p* = 0.04). SSRIs may modulate preeclampsia risk and mechanisms, although further studies are needed to investigate the relationships between 5-HT and AVP in depression and preeclampsia.

## Introduction

Approximately 14–23% of pregnant women experience depression symptoms during pregnancy. However, only 14% of these women receive treatment [1, 2]. Those with depression are at 1.63 times higher odds of developing preeclampsia (PreE) [3]. PreE, a hypertensive disease of pregnancy, affects 5–8% of all US pregnancies (400,000/year) and is a leading cause of worldwide obstetric mortality, causing 76,000 maternal and 500,000 neonatal deaths annually [4, 5]. Notably, women who are diagnosed with PreE are at 2.4 times higher odds of developing depression [6].

While depression and PreE are associated, mechanisms linking this association remain elusive. One potential mechanism may involve arginine vasopressin (AVP) which affects stress, vascular, immune, and osmotic responses in the body [4] and plays a role in depression symptoms [7]. Other groups have demonstrated that AVP is elevated and is important in the etiology of major depressive disorder [8–10]. We and others have shown that in patients who develop PreE, elevated AVP secretion (as measured by levels of copeptin) is detected throughout pregnancy and is robustly predictive of the disease [4, 11–20]. Copeptin is produced in a 1:1 ratio with AVP. It is a surrogate measure of AVP because it is more reliably measured in assays [21]. In a pregnant mouse model utilizing chronic, low-dose AVP administration throughout gestation, human PreE phenotypes are replicated including pro-inflammation, endothelial dysfunction, abnormal placentation, pregnancy-specific maternal hypertension, renal dysfunction, and fetal growth restriction [20, 22, 23]. In addition, animal models reveal elevated AVP with glucocorticoid-mediated stress [24]. AVP may also be a potential biomarker for the efficacy of depression treatment efficacy [25]. Taken together, these data indicate that AVP dysregulation is a common link between the pathogenesis of PreE and depression.

A further link between depression and PreE may involve dysregulation of serotoninergic systems, which are tightly coupled in the central nervous system to vasopressin secretion and in the immune system to inflammatory factor production [26, 27]. Placental serotonin (5-HT) receptor expression [28, 29], maternal and uterine artery vasoreactivity to serotonin [30], and circulating serotonin concentration are all abnormal in PreE [31–33]. Placental and circulating 5-HT are similarly dysregulated in depression [33, 34].

Given strong regulatory links between the serotonergic and vasopressin systems, others studied whether Selective Serotonin Reuptake Inhibitors (SSRIs) might be PreE-protective. While one large study on SSRI monotherapy for depression demonstrated no increased risk for PreE (RR: 1.00, 95% CI: 0.93–1.07) [35], others have found an increased risk for the disease [36–38]. However, these epidemiological studies fail to capture the impact of additional patient comorbidities, mood disorder severity, and effects of molecular mediators [39, 40]. The objective of the present study was to investigate the associations among AVP secretion, depression symptoms, SSRIs, and preeclampsia during pregnancy to reveal the molecular basis for clinical depression-PreE links.

## Materials and Methods

### Cohort Assembly

**Fig. 1:**
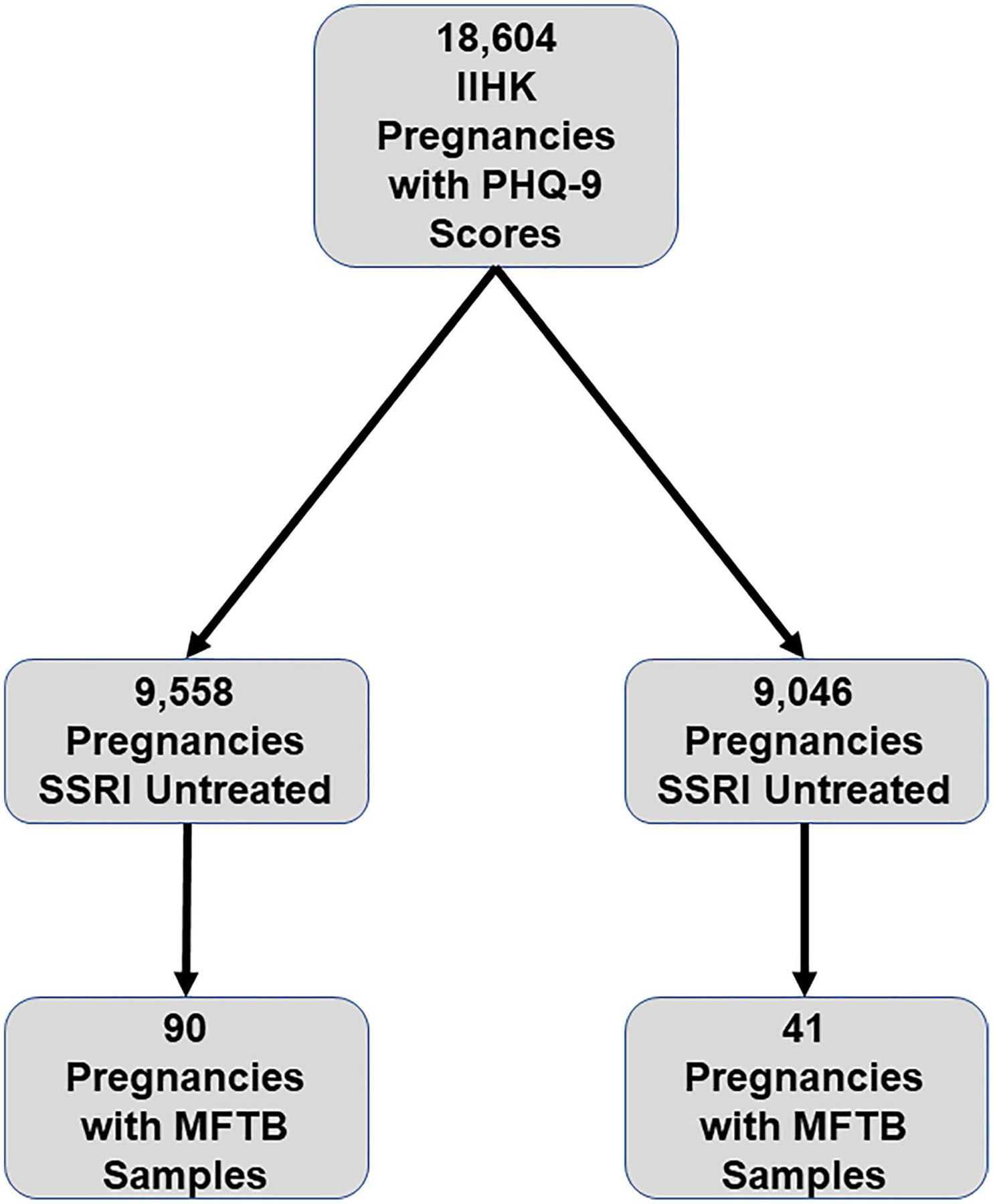
Assembly of cohorts. All pregnancies analyzed were represented in the Iowa Intergenerational Health Knowledgebase

The overall objective of this study was to evaluate the relationship between SSRI exposure in pregnancy, PreE, depression, and circulating copeptin levels. To this end, a retrospective cohort dataset was constructed using the Iowa Intergenerational Health Knowledgebase (IIHK). This study was IRB approved (IRB # 201,710,819). The IIHK is an OB/Gyn and Pediatrics enterprise data warehouse. All clinical data regarding pregnancies and maternal-child dyads are captured from the electronic health record (Epic) and retrieved from IIHK. As depression symptoms are a significant confounding variable in this study which may mediate both SSRI use and PreE risk, it was important to identify pregnancies within the IIHK with documented Patient Health Questionnaire (PHQ)-9 scores. The PHQ-9 is a validated, commonly used screening tool for depression symptoms in pregnancy [41, 42]. Within the IIHK, 18,604 pregnancies had PHQ-9 scores available. SSRI exposure in pregnancy was defined as the existence of an SSRI (citalopram, escitalopram, fluoxetine, paroxetine, and sertraline) on the active medication record for a particular pregnancy. Of the 18,604 pregnancies, PHQ-9 scored pregnancies, 9558 were SSRI untreated and 9046 were SSRI treated (Fig. 1). Data from these scored pregnancies were used in the analyses.

A nested study was conducted to (1) investigate if the separate and combined effects of depression symptoms and SSRI use significantly dysregulate early pregnancy (< 20 weeks) maternal plasma copeptin levels and (2) determine if SSRI-related copeptin dysregulation is associated with altered PreE risk. The Maternal Fetal Tissue Bank (MFTB) [IRB #200910784] at the University of Iowa (UI) is a cross-sectional, prospectively collected biorepository where samples and clinical data are obtained from women who provide informed consent, coded, and uniformly processed, as described previously [43]. The MFTB includes maternal blood and urine collected longitudinally throughout pregnancy, cord blood, and placental tissue, as well as information on the long-term health of mothers and children [43]. Given the use of coded clinical data and biosamples provided by the MFTB, the UI IRB determined that the study is not considered human subjects research. Of the 5248 MFTB pregnancies, 727 had first trimester PHQ-9 scores, of which 233 also had first trimester maternal plasma samples available.

### Procedures

#### Inclusion/Exclusion Criteria

Minimum maternal inclusion criteria include (1) age 18 or older, (2) English speaking, (3) pregnant, and (4) able to give informed consent. Exclusion criteria are any of the following: (1) hepatitis C positive, (2) ward of the court (mother or child), or (3) HIV positive. Preeclampsia cases are defined per standard American College of Obstetricians and Gynecologists (ACOG) definitions and validated by the MFTB research team [44].

#### Demographic and Clinical Data

Demographics included age, race/ethnicity, insurance type, and marital or partner status. Maternal characteristics included gravida status, body mass index (BMI), chronic essential hypertension, chronic pain condition, past mental history (ICD-10 codes for depression and anxiety), current or history of PreE, pre-existing diabetes mellitus, SSRIs, and copeptin levels. Pregnancy characteristics include gestational age at delivery, vaginal or C-section delivery, newborn birth weight, and 1- and 5-min APGAR scores.

#### Copeptin Measurements

All early pregnancy (< 20 weeks) maternal plasma copeptin concentrations were measured in duplicate via a commercially available enzyme-linked immunosorbent assay (ELISA) (USCN Life Science, Inc.), as previously described by our group [20]. Copeptin was normalized to total plasma protein, which was measured by a commercial bicinchoninic acid assay (Thermo Scientific) [20].

#### Perinatal Depression Measure

The Patient Health Questionnaire (PHQ-9) is a self-administered depression severity measure that is administered during pregnancy to all participants during routine clinical care. Depression symptom severity is categorized by PHQ-9 score (PHQ 0–3 = no-to-minimal symptoms, 4–9 = mild symptoms, 10–14 = moderate symptoms, ≥ 15 = severe symptoms) [45]. The Cronbach’s alpha for the PHQ-9 is excellent at 0.89 [42, 45]. Criterion-related validity indicates a mid-level correlation (*r* = 0.77, *p* ≤ 0.001) with the Perinatal Depression Inventory (PDI) [46]. A cutoff of 0.7 indicates a PHQ-9 sensitivity and specificity of 81% and 79%, respectively [45, 47].

#### Statistical Analyses

Data analyses were performed using SPSS, version 25.0 (IBM Corp, 2017). As appropriate, T-tests or chi-square were used to examine differences in covariates between groups. Multivariable logistic regression was performed to evaluate associations between depression and SSRI use with PreE as the dependent variable. ANOVA was performed to evaluate the relationship of SSRI treatment and preeclampsia on copeptin levels. Published early pregnancy (< 20 weeks) maternal plasma copeptin levels [20, 48] demonstrate a copeptin concentration effect size of 100 pg/mL to detect a difference between controls and preeclamptics. To detect a difference in the current study, assuming a similar effect size in copeptin and 80% power and alpha = 0.5, a minimum of 20 participants with measured copeptin would be needed.

## Results

### Controlled Analyses Reveal Decreased Risk of Preeclampsia Among SSRI Users

Initially, we used the large IIHK retrospective clinical cohort (Fig. 1, *N* = 18,604) to evaluate differences in gestational risk and characteristics among SSRI-treated and untreated mothers. There were no clinically significant differences in mean maternal age, mean body mass index (BMI), racial distribution, median gravidity, and median parity between SSRI-treated and untreated groups (Table 1). In uncontrolled analyses, there were significantly slightly higher rates of chronic hypertension (15% vs 12%, *p* < 0.001), PreE (10% vs 8%, *p* < 0.001), diabetes (16% vs. 13%, *p* < 0.001), and multiple gestation (55% vs. 44%, *p* < 0.001) in the SSRI-treated group versus the untreated group. As expected, SSRI-treated mothers also had slightly higher rates of diagnosed depression (30% vs. 27%, *p* < 0.001) and anxiety (34% vs. 30%, *p* < 0.001) than SSRI-untreated mothers. As hypothesized, when controlled for severity of depression symptoms and other maternal characteristics (as in Table 2), SSRI treatment demonstrated a significantly lower risk of preeclampsia. In all logistic regression models noted in Table 2, SSRI exposure was significantly associated with a lower risk of preeclampsia (OR = 0.8–0.9) and increased depression severity was associated with a higher risk of preeclampsia (OR = 1.1–1.2). Even after for controlling for maternal BMI, maternal age, chronic hypertension, and diabetes, this relationship of a lower risk of preeclampsia with SSRI treatment is persistent (Table 2, Model 4, OR = 0.9 (0.7–1.0), *p* = 0.05).

### SSRIs are Preeclampsia Protective in Well-controlled Depression

**Fig. 2:**
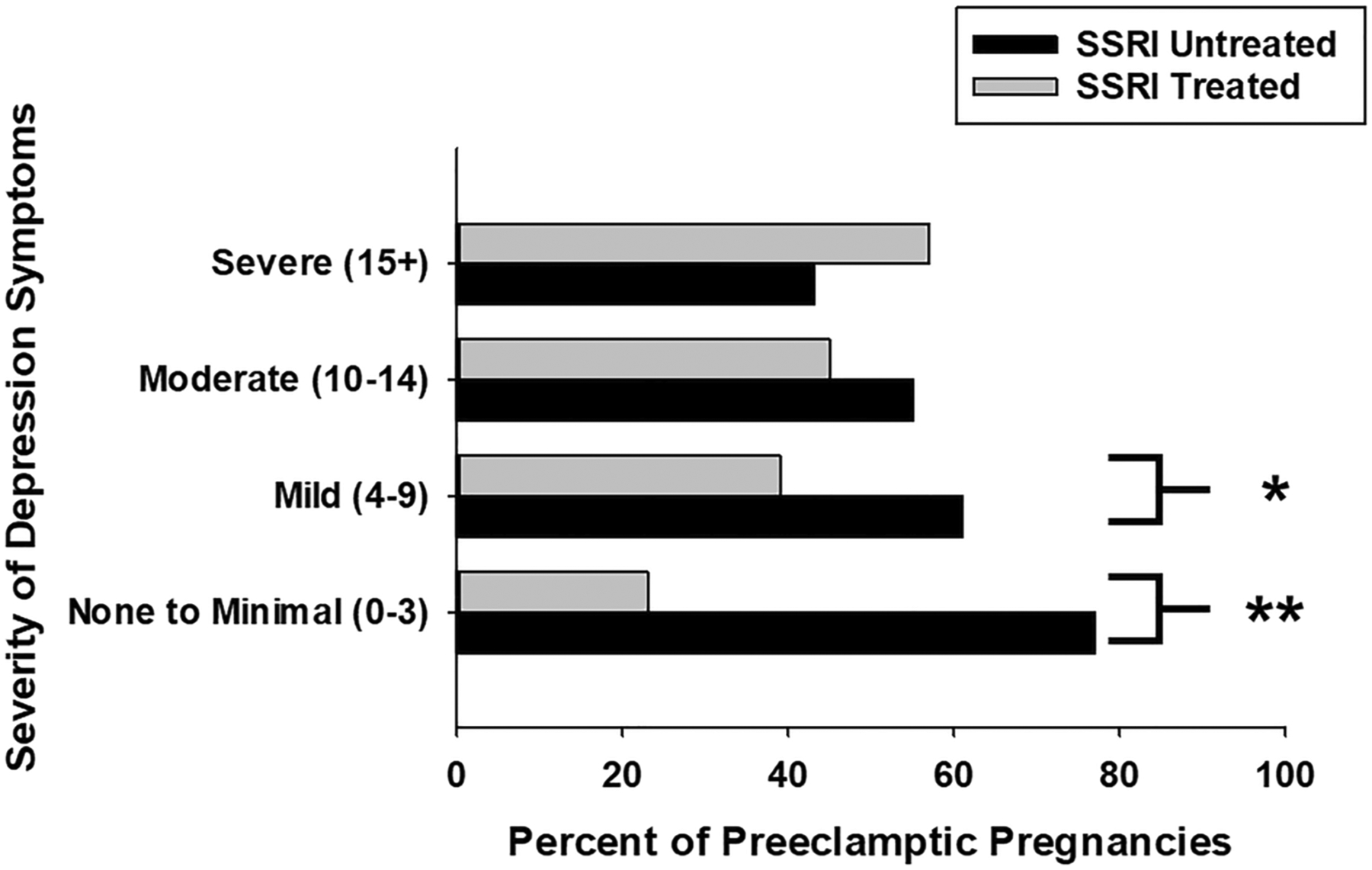
Pregnant women affected by preeclampsia with no-to-mild depression symptoms exhibit lower rates of SSRI treatment in the IIHK cohort. In the large Iowa Intergenerational Health Knowledgebase cohort, preeclamptic women with no (PHQ 9 = 0–3, 23% vs. 77%, **p = 0.010) to mild (PHQ 9 = 4–9, 39% vs. 61%, *p = 0.04) depression symptoms exhibit significantly lower rates SSRI treatment. This relationship was not observed in those with moderate-to-severe depression symptoms

To further understand whether SSRIs might have differential impacts on those with varying severities of depression, we further investigated the relationship between SSRI treatment, the severity of depression symptoms, and PreE. In the setting of no (PHQ 9 = 0–3, 23% vs. 77%, *p* = 0.010) and mild (PHQ 9 = 4–9, 39% vs. 61%, *p* = 0.04) depression symptoms, there were significantly fewer preeclamptic patients who were SSRI treated (Fig. 2). Rates of SSRI treatment were similar in those women with preeclampsia and moderate (PHQ 9 = 10–14, 45% vs. 55%, *p* = 0.3) and severe (PHQ 9 > 14, 57% vs. 43%, *p* = 0.5) depression symptoms (Fig. 2). Together, these data demonstrate that in pregnant women with mild or no depression symptoms, but not those with poorly controlled (moderate or severe) depression, SSRI treatment was preeclampsia protective.

### Vasopressin Secretion is Increased in Pregnancies Affected by Depression and is Attenuated by SSRIs

To investigate the association between vasopressin and serotonin in mood disorders and PreE, we measured copeptin in a smaller, nested cohort (with SSRI-treated and untreated subsets) of pregnancies for which early pregnancy PHQ-9 scores and maternal plasma samples were available from the MFTB.

**Fig. 3:**
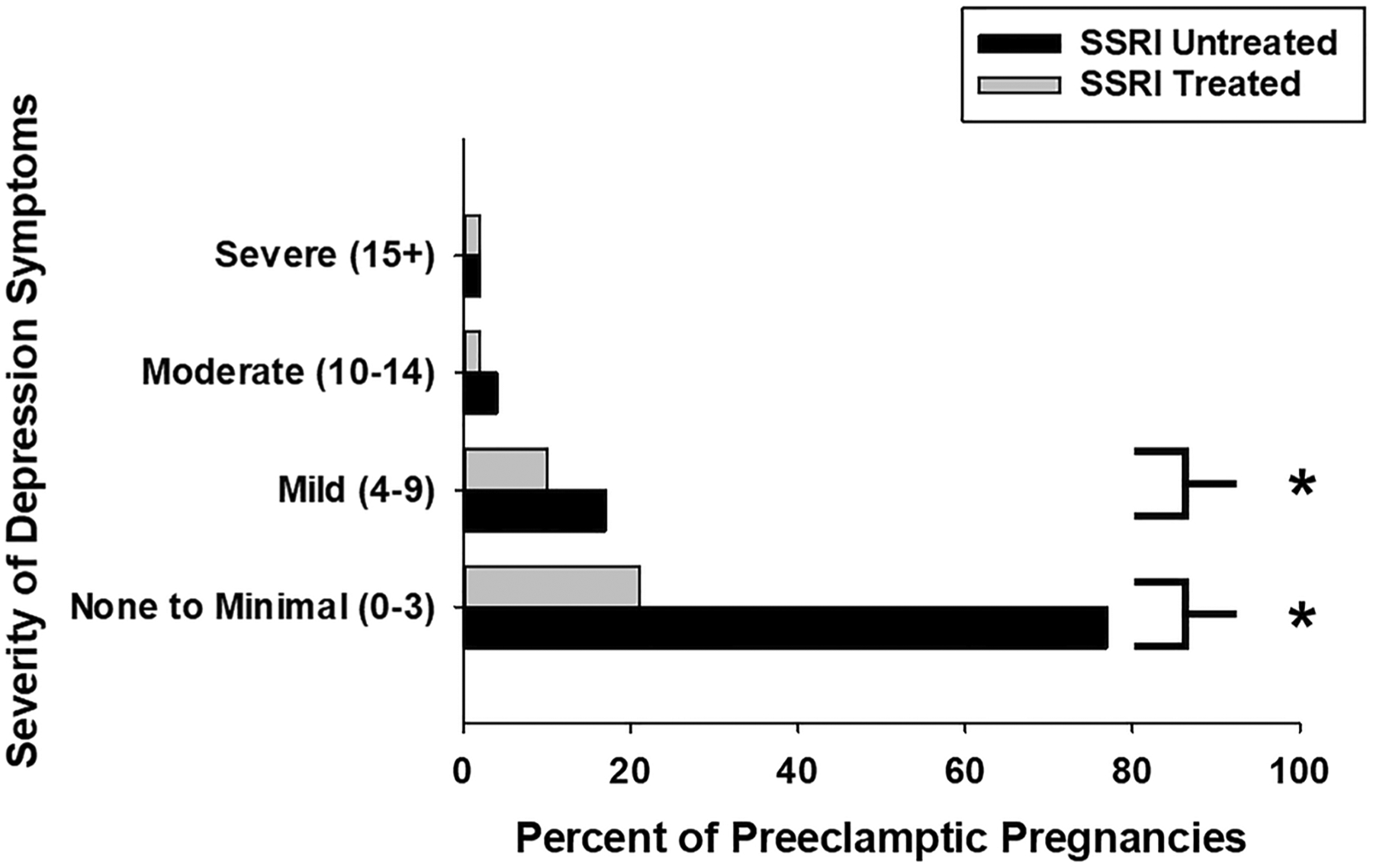
Pregnant women affected by preeclampsia with no-to-mild depression symptoms exhibit lower rates of SSRI treatment in a nested MFTB cohort. Consistent with the findings in the IIHK cohort, in a nested cohort for whom biosamples were available, preeclamptic women with no (PHQ 9 = 0–3, 21% vs. 77%, *p < 0.001) to mild (PHQ 9 = 4–9, 10% vs. 17%, *p < 0.001) depression symptoms exhibit significantly lower rates of lower rates SSRI treatment. This relationship was not observed in those with moderate-to-severe depression symptoms

In a sensitivity analysis, this nested MFTB cohort used for the measurement of copeptin demonstrated a similar distribution of maternal characteristics (age, BMI, race, gravida, parity) and maternal comorbidities (chronic hypertension, preeclampsia, diabetes, multiple gestations, and mood disorder diagnoses) in SSRI-treated and untreated groups as the larger parent retrospective cohort of 18,604 patients (Online Resource 1). This nested cohort was representative of our finding in the larger cohort (Fig. 1) that significantly lower rates of SSRI treatment were found in preeclamptic pregnancies with no (PHQ 9 = 0–3, 21% vs. 77%, *p* < 0.001) or mild (PHQ 9 = 4–9, 10% vs. 17%, *p* < 0.001) depression symptoms, but not in those with poorly controlled depression (PHQ 9 = 10–15 +) (Fig. 3).

**Fig. 4:**
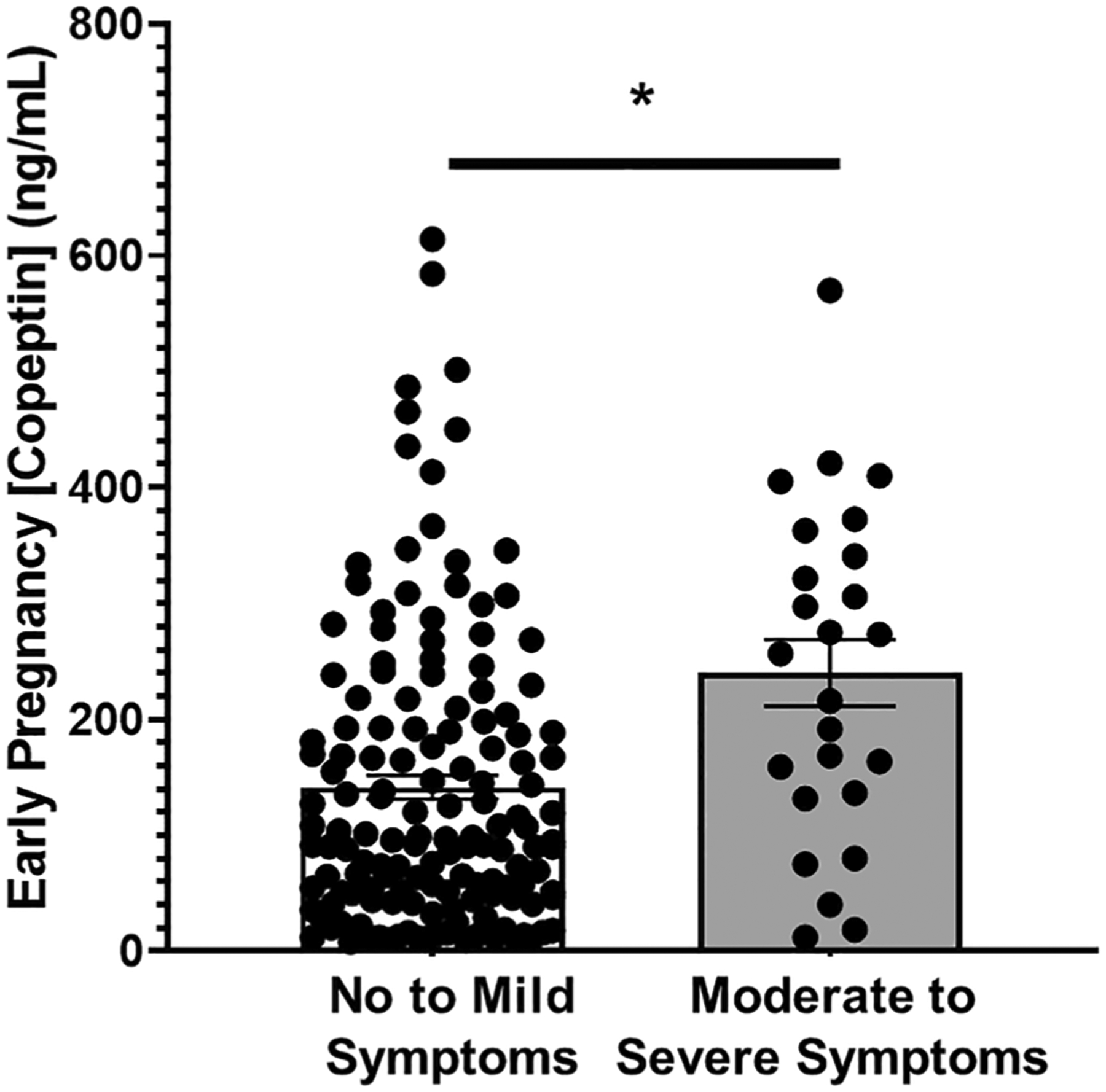
Moderate-to-severe depression symptoms are associated with elevated early pregnancy (< 20 weeks) plasma copeptin. Pregnant women with moderate-to-severe depression symptoms and a Patient Health Questionnaire (PHQ-9) score of ≥ 10 exhibit a significantly higher mean first trimester plasma copeptin in comparison to those with no-to-mild depression symptoms (240 ± 29 vs. 142 ± 10 ng/mL, *p < 0.001)

**Fig. 5:**
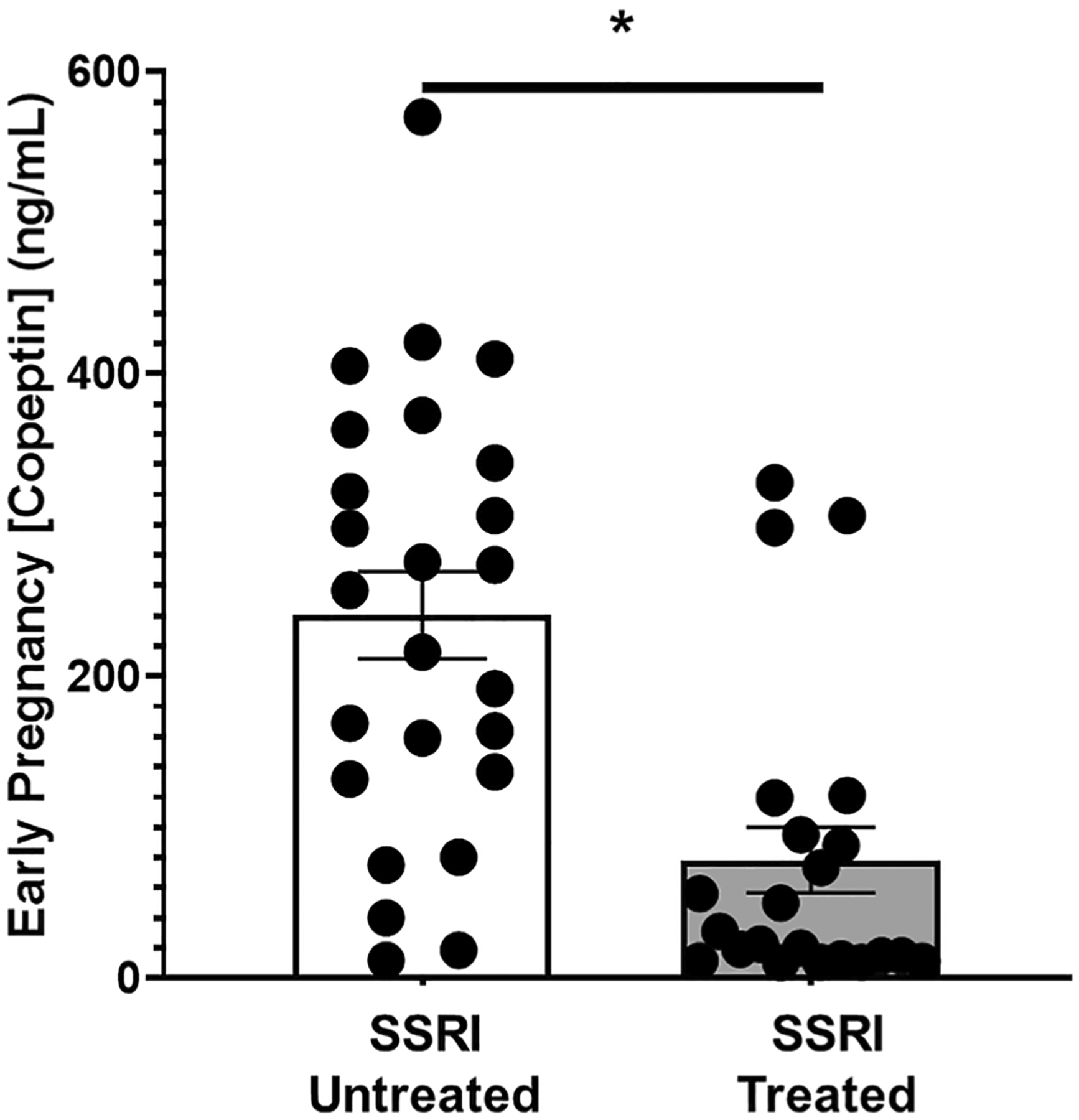
Selective serotonin reuptake inhibitor use in pregnancy is negatively associated with early pregnancy (< 20 weeks) plasma copeptin. Pregnant women using Selective Serotonin Reuptake Inhibitors (SSRIs) in pregnancy exhibit significantly decreased mean first trimester plasma Copeptin (78 ± 22 vs. 240 ± 29 ng/mL, *p < 0.001)

In this nested cohort, we found a significant positive association between copeptin levels and depression symptoms, indicating increased vasopressin signaling (as occurs in preeclampsia) in those with moderate-to-severe depression symptoms. Pregnant women with moderate-to-severe depression symptoms exhibited significantly higher mean early pregnancy copeptin than those with mild-to-no depression symptoms (Fig. 4, 240 ± 29 [*n* = 25] vs. 142 ± 10 [*n* = 148] ng/mL, *p* < 0.001). Furthermore, we found that SSRIs significantly lowered plasma copeptin in pregnant women with depression symptoms (Fig. 5, 78 ± 22 SSRI medicated [*n* = 25] vs. 240 ± 29 [*n* = 22] SSRI unmedicated, ng/ml, *p* < 0.001).

### SSRIs Decrease the Causative Preeclampsia Biomarker, Vasopressin secretion, in Moderate and Severe Depression.

In women with moderate and severe depression, who are known to be at increased risk for preeclampsia [3, 49, 50], we investigated SSRI modulation of the causative preeclampsia biomarker, vasopressin secretion. Vasopressin secretion, measured by plasma copeptin, is an early pregnancy biomarker and a causative risk factor for preeclampsia [20].

**Fig. 6:**
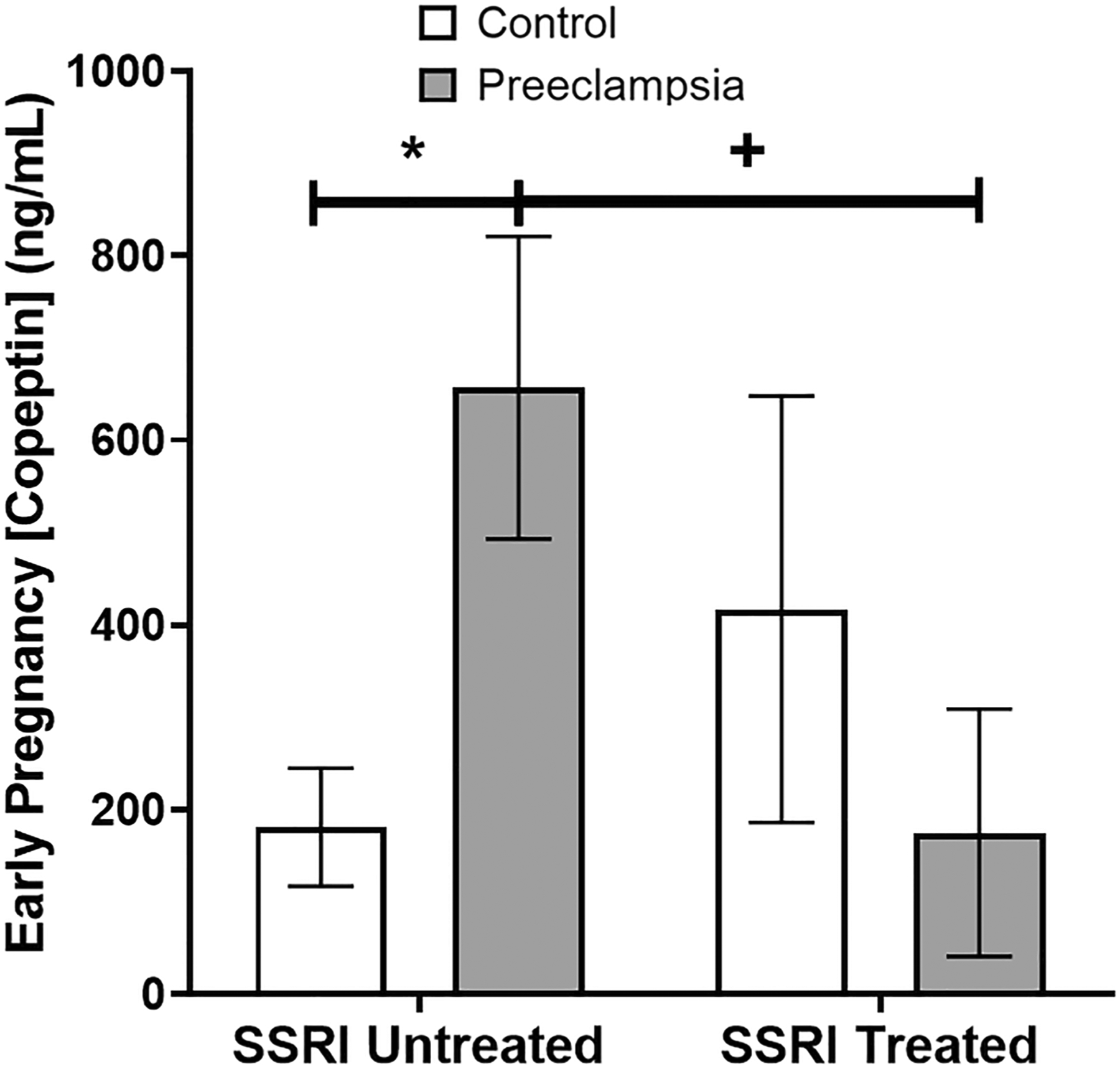
SSRI treatment is associated with decreased plasma copeptin in women with preeclampsia. In women with moderate-to-severe depression symptoms, there is a significant increase in copeptin in SSRI-unmedicated, preeclampsia-affected pregnancies in compared to controls (657 ± 164 vs. 181 ± 64 ng/mL, *p* = 0.02). In pregnancies affected by preeclampsia, SSRI treatment is associated with significantly lower copeptin levels (657 ± 164 vs. 175 ± 134 ng/mL, *p* = 0.04). Notably, the interaction of SSRI treatment and preeclampsia was also significant by ANOVA (*p* = 0.04)

In women with moderate-to-severe depression symptoms and who were SSRI unmedicated, copeptin was significantly increased in preeclamptic pregnancies (Fig. 6, 657 ± 164 [*n* = 10] vs. 181 ± 64 ng/mL [*n* = 10], *p* = 0.02). Critically, this preeclampsia-associated increase in copeptin was not seen in women with moderate-to-severe depression who were SSRI medicated. Furthermore, in pregnancies affected by preeclampsia, SSRI treatment was associated with significantly lower copeptin levels (Fig. 6, 657 ± 164 [*n* = 10] vs. 175 ± 134 ng/mL [*n* = 10], *p* = 0.04). Of note, there is a significant interaction term (2-way ANOVA, *p* = 0.04) with regard to SSRI treatment and preeclampsia effects on copeptin.

### SSRI Treatment Does Not Broadly Alter Pregnancy Outcomes

Finally, we evaluated the effect of SSRI use on clinically significant pregnancy outcomes in the large retrospective cohort. While there were some statistical differences, we found no clinically significant difference in mean gestational age at delivery, cesarean section rate, mean birthweight, or median APGAR scores between the SSRI-untreated and treated groups (Table 3).

## Discussion

The present study of clinical and molecular associations between depression symptoms, SSRI use, and preeclampsia led to several conclusions. First, in a large clinical dataset, we demonstrate that less preeclamptic women receive SSRI treatment. This association is not observed in those with moderate-to-severe depression symptoms. Even after controlling for significant clinical covariates, SSRI exposure in pregnancy is associated with a lower risk of preeclampsia. As a marker of AVP secretion, we demonstrate that maternal plasma copeptin at less than 20 weeks of gestation was higher in women with moderate-to-severe depression symptoms in comparison to those with no-to-mild symptoms. In general, we find that SSRI treatment is associated with lower copeptin levels. Specifically, in those with more severe depression symptoms, copeptin levels are significantly lower in SSRI-treated pregnant women affected with preeclampsia. By stratifying by depression severity, we detect a depression severity-sensitive relationship between copeptin (vasopressin secretion), SSRI use, and preeclampsia. These data indicate that SSRI use in pregnancy may be protective against the development of preeclampsia, both at the level of diagnosis and at the level of the clinically significant biomarker, vasopressin. This protective effect may be further mediated by the effect of SSRI exposure on AVP secretion, with more well-controlled depression revealing little amelioration of preeclampsia risk, while more poorly controlled (moderate or severe depression) saw significant benefits of SSRI use on vasopressin release.

In light of these findings, we posit that the preeclampsia-protective effect of SSRI therapy is associated with decreased peripheral AVP (Graphical Abstract). Our data further support a potential mechanism by which AVP is attenuated by 5-HT, thereby decreasing preeclampsia risk in the setting of poorly controlled, severe depression. The magnitude of serotonin dysregulation, and the efficacy of SSRIs, is associated with depression severity [34, 51–53]. AVP secretion (as assayed by circulating copeptin) is a mechanistically interesting link between the serotonergic disorders of depression and preeclampsia. Early and tonic elevations of AVP secretion have been (1) associated with human preeclampsia [4, 11–20] and (2) are sufficient to replicate preeclampsia-like phenotypes in mice [20, 22, 23, 54]. In pregnant women with depression symptoms, we found here that circulating AVP secretion is elevated. Yet, we observe a lower risk for preeclampsia rates in women treated with SSRIs. This finding contradicts current literature demonstrating no increased risk for preeclampsia [35] or increased risk with SSRI use in pregnancy [36–38]. Notably, this study controlled for the severity of depression symptoms and found that SSRI treatment is associated with a lower risk of preeclampsia. It is possible that underlying depression, rather than SSRI use itself, drives the risk for preeclampsia in prior work [55]. The use of a mechanistically relevant biomarker, copeptin, in the study presented here allows for some disentangling of the relationship between preeclampsia and depression.

As a result of this study, we hypothesize that elevated AVP is a molecular link between depression and preeclampsia and indicates a potential therapeutic pathway to target. In total, the findings presented here demonstrate that SSRIs may be preeclampsia preventative. Furthermore, our copeptin data support the conclusion that SSRI efficacy for use in preeclampsia may be supported by physiologic interactions between AVP and serotonin.

## Clinical and Research Implications

Multiple biological mechanisms may underlie the downregulation of copeptin/vasopressin by SSRI therapy. While our data evaluate circulating levels of copeptin, central neurologic mechanisms support co-regulatory processes of AVP and serotonin. Direct stimulation of AVP neurons via V_1A_ receptors increases dorsal raphe serotonin neuron firing rates [26]. Furthermore, these dorsal raphe serotonin neurons directly downregulate AVP neuron activity in the supraoptic nucleus [24, 26, 56]. In fact, the SSRI fluoxetine modulates plasma AVP and pituitary AVP receptor expression [26]. Additional findings in animal models also demonstrate cross-talk between vasopressin and serotonin systems at the behavioral level, with V_1b_ receptor antagonism achieving anti-depressant-like effects [57] and SSRI rescue of depression-like phenotypes acting in an AVP-dependent fashion [26, 58]. Our findings support the notion that serotonin and AVP systems interact during human preeclampsia via SSRI-dependent mechanisms to modulate AVP secretion, as measured by peripheral levels of the AVP prohormone copeptin.

SSRI interactions with central nervous system networks are also dependent on the serotonin transporter (SERT), which is unable to efficiently uptake serotonin from the extracellular environment when bound by an SSRI. Though contested by some, it is broadly understood that SERT expression, function, and activity are related to depression and mood symptoms, as well as SSRI efficacy [59, 60]. While expression studies suggest levels of SERT may be unchanged in the preeclamptic placenta [61], the field has not yet examined whether SERT disruptions at the genomic or functional levels might predispose individuals to risk for gestational hypertension. Disruptions to SERT expression or function in preeclampsia may impact placental sequestration of serotonin, thereby altering its availability to the fetus and/or placental vessels. This is therefore an important avenue for further exploration.

These results suggest that women at high risk for preeclampsia may benefit from SSRI therapy as those women are also at risk for depression [38]. The risks of untreated depression in pregnancy can be severe, however, and include increased rates of preeclampsia, preterm labor, low birth weight, and intrauterine growth restriction [62]. During pregnancy, only 26% of women continue their antidepressants despite SSRIs being considered safe and well-tolerated in pregnancy [63, 64].

In addition to revealing a potential preventative or therapeutic role for SSRIs in preeclampsia, the present study also highlights a potential biomarker. To better prevent preeclampsia, improved detection of women at increased risk using serotonin dysregulation and copeptin as biomarkers may be useful. Up to 70% of pregnant women experience depression symptoms, while only 10–16% of these meet a diagnostic threshold for depression [2, 65, 66]. In addition, at least 20% of women screened do not disclose depression symptoms due to stigma, time constraints, and lack of motivation [67]. Biomechanistic and microarray analyses of preeclampsia markers, genes, and an immunologic presentation subtype may further help to identify whether copeptin is decreased in distinct molecular subsets of women with preeclampsia and/or depression [68–70].

### Strengths and Weaknesses

The current study benefitted from high-quality biosamples and clinical data from the Maternal–Fetal Tissue Bank and the Iowa Intergenerational Health Knowledgebase [43]. There is strong fidelity in biosample processing and storage to maintain sample quality and integrity. The veracity of the retrospective clinical data used here is powered by the OBstetrics Data Integration Architecture (OBDIA) structure of the Iowa Intergenerational Health Knowledgebase. The OBDIA allows for the integration of multiple data sources within the institution, including, but not limited to, imaging data, diagnoses, vital sign information, medications, medication administration, and procedures for the maternal–fetal dyad. In addition, clinical outcomes of interest such as preeclampsia, are validated by content experts after cases are identified by their respective diagnosis codes.

Furthermore, the present study was appropriately powered to evaluate the desired outcomes. One weakness is the predominantly homogenous sample population with > 70% of the cohort population identified as White. Yet, this racial distribution is consistent with the current population of Iowa [71]. While studies such as this one must be validated in a more diverse population, the conclusions of this study applied to approximately 30% of the cohort population who were non-White. This observation suggests that vasopressin and serotonin dysregulation in preeclampsia and depression may apply more generally. Due to sample limitations, we were unable to determine serotonin levels in the cohort. Future studies should examine whether free systemic, placental, and platelet serotonin are disrupted in coordination (e.g., correlated or anti-correlated) with vasopressin release to further illucidate serotonin-vasopressin interactions in preeclampsia and in normal pregnancy. Other sources of bias may include confounding variables for which we are underpowered to analyze, such as other concomitant therapy (e.g., psychotherapy) [72].

## Conclusions

Despite a clear association between depression and preeclampsia, the mechanistic interplay between depression and preeclampsia is complex and poorly understood. The novel findings presented here support the hypothesis that AVP secretion, as measured by copeptin, is modulated by SSRI use in women with depression symptoms, a population that is at increased risk for preeclampsia [73]. We further find that decreased AVP secretion with SSRI therapy may decrease preeclampsia risk in those with poorly controlled depression. Ongoing studies in early pregnancy will continue to quantify and characterize the relationship and biomechanisms mediating the relationship between serotonin dysregulation and preeclampsia risk.

## Supplementary Material

Supplementary Material

## Figures and Tables

**Table 1:** Maternal child knowledgebase cohort characteristics

	SSRI untreated (*N* = 9558)	SSRI treated (*N* = 9046)	*P* value
Age (years)	31 ± 9	31 ± 8	0.4
Body mass index (kg/m^2^)	30 ± 7	30 ± 8	0.3
White (%)	75%	73%	0.1
Gravida (*N*)	2 (1–4)	2 (1–3)	0.005
Para (*N*)	1 (0–2)	1 (0–2)	0.01
Chronic hypertension (%)	12%	15%	< 0.001
Preeclampsia (%)	8%	10%	< 0.001
Diabetes (%)	13%	16%	< 0.001
Multiple gestations (%)	44%	55%	< 0.001
Depression (%)	27%	30%	< 0.001
Anxiety (%)	30%	34%	< 0.001
PHQ = 0–3 None to minimal (%)	77%	21%	0.01
PHQ = 4–9 Mild (%)	17%	10%	0.04
PHQ = 10–14 Moderate (%)	4%	8%	0.3
PHQ ≥ 15 Severe (%)	1%	3%	0.5

Age and body mass index are presented as means ± standard deviation. Gravidity and parity are presented as median (25–75th percentile). Categorical variables are presented as percentages. Alpha = 0.05

**Table 2:** Regression modeling

Models and independent variables	Odds ratio (95% CI)	*P* value
Model 1		
SSRI exposure	0.8 (0.7–0.9)	< 0.001
Depression severity	1.2 (1.1–1.3)	0.002
Model 2: model 1 + maternal characteristics		
SSRI exposure	0.8 (0.7–0.9)	0.002
Depression severity	1.1 (1.0–1.2)	0.05
Body mass index	1.1 (1.0–1.1)	< 0.001
Maternal age	1.0 (0.9–1.0)	0.3
Model 3: model 1 + maternal comorbidities		
SSRI exposure	0.8 (0.7–0.9)	0.007
Depression severity	1.1 (1.0–1.2)	0.04
Chronic hypertension	4.0 (3.4–4.8)	< 0.001
Diabetes	1.6 (1.3–1.8)	< 0.001
Model 4: model 1 + maternal characteristics and comorbidities		
SSRI exposure	0.9 (0.7–1.0)	0.05
Depression severity	1.1 (1.0–1.2)	0.1
Body mass index	1.0 (1.0–1.0)	< 0.001
Maternal age	1.0 (0.9–1.0)	0.01
Chronic hypertension	3.8 (3.3–4.5)	< 0.001
Diabetes	1.5 (1.3–1.8)	< 0.001

Preeclampsia diagnosis is the dependent variable in all models. Alpha = 0.05

**Table 3:** Pregnancy outcomes in the Iowa Intergenerational Health Knowledgebase cohort

	SSRI untreated (*N* = 9558)	SSRI treated (*N* = 9046)	*P* value
Gestational age at delivery (days)	270 ± 148	272 ± 158	0.5
C-section	31%	33%	0.02
Birthweight (g)	3307 ± 661	3255 ± 687	< 0.001
APGAR 1	8 (7–9)	8 (7–9)	< 0.001
APGAR 5	9 (9–9)	9 (8–9)	< 0.001

Gestational age at delivery and birthweight are presented as means ± standard deviation. APGAR scores at 1 min and 5 min are presented as median (25–75th percentile). Categorical variables are presented as percentages. Alpha = 0.05

## Data Availability

All data are available upon reasonable request from the authors.
