# Supplementary material for: Selective Serotonin Reuptake Inhibitor Use in Pregnancy and Protective Mechanisms in Preeclampsia
Source: Reprod Sci. 2022 Aug 19;30(2):701–12. doi: 10.1007/s43032-022-01065-z (PMC9944568; doi:10.1007/s43032-022-01065-z)
Supplement: Supplementary file 1 — Supplementary file1 (DOCX 20 KB) [file 43032_2022_1065_MOESM1_ESM.docx]

**Online Resource 1:**

**Maternal Fetal Tissue Bank Nested Cohort Characteristics**

|  | SSRI Untreated  N=90 | SSRI Treated  N=41 | P Value |
| --- | --- | --- | --- |
| Age (years) | 31 ± 5 | 30 ± 5 | 0.5 |
| Body Mass Index (kg/m^2^) | 28 ± 1 | 31 ± 1 | 0.01 |
| White (%) | 81% | 80% | 0.1 |
| Gravida (N) | 3 (1-3) | 2 (1-3) | 0.2 |
| Para (N) | 1 (0-2) | 0 (0-2) | 0.02 |
| Chronic Hypertension (%) | 14% | 29% | 0.07 |
| Preeclampsia (%) | 29% | 4% | <0.001 |
| Diabetes (%) | 71% | 29% | 0.8 |
| Multiple Gestation (%) | 0% | 5% | 0.2 |
| Depression (%) | 27% | 81% | < 0.001 |
| Anxiety (%) | 21% | 79% | < 0.001 |
| PHQ=0-3  None to Minimal (%) | 77% | 21% | < 0.001 |
| PHQ = 4-9  Mild (%) | 17% | 10% | < 0.001 |
| PHQ = 10-14  Moderate (%) | 4% | 2% | 0.3 |
| PHQ ≥ 15  Severe (%) | 2% | 2% | 0.4 |
| Copeptin (ng/mL) | 240 ± 29 | 78 ± 22 | < 0.001 |

Age and body mass index are presented as means ± standard deviation. Gravidity and parity are presented as median (25-75 percentile). Categorical variables are presented as percentages. Alpha=0.05
